# ATP-gated P2X receptors in health and disease

**DOI:** 10.3389/fncel.2014.00204

**Published:** 2014-07-24

**Authors:** Geoffrey Burnstock, Andrea Nistri, Baljit S. Khakh, Rashid Giniatullin

**Affiliations:** ^1^Division of Biosciences, Autonomic Neuroscience Centre, University College Medical SchoolLondon, UK; ^2^Department of Pharmacology and Therapeutics, The University of MelbourneParkville, VIC, Australia; ^3^Neuroscience Department, International School for Advanced Studies (SISSA)Trieste, Italy; ^4^Department of Physiology, David Geffen School of Medicine, University of California, Los AngelesLos Angeles, CA, USA; ^5^Department Neurobiology, A. I. Virtanen Institute, University of Eastern FinlandKuopio, Finland; ^6^Laboratory of Neurobiology, Department of Physiology, Kazan Federal UniversityKazan, Russia

**Keywords:** ATP, P2X receptor, ion channels, neurotransmitters, central nervous system

This collection is devoted to the role of extracellular adenosine 5′-triphosphate (ATP) as a physiological transmitter in health and as an essential contributor to various pathologies. The present e-book contains 15 reviews (and 2 original papers) detailing the action of extracellular ATP on the peripheral and central nervous systems. Thus, this e-book provides a major repository of novel information on ATP-gated P2X receptors that are important transducers of the cellular effects of ATP.

ATP has long been known as the main source of energy in living cells. However, a further fundamental role of ATP was later identified, namely that extracellular ATP serves as a messenger for fast intercellular communications via binding to and activation of a set of membrane proteins termed P2X receptors. Unlike other classical neurotransmitters and neuromodulators, the extracellular actions of ATP are pleiotropic as they can affect almost all cell types (both neuronal and non-neuronal) in the body. Thus, ATP has rightly joined “the club” of traditional signaling molecules and is now considered to be a phylogenetically early and widely distributed endogenous agonist. The history and perspectives for this subject are presented in the opening paper “Introduction and perspective, historical note” written by Geoffrey Burnstock, the founding father of the field (Burnstock, 2013). The evolutionary thread of P2X receptors found in primitive organisms is discussed by Fountain (2013).

In the nervous system, ATP-gated P2X receptors are expressed in neurons, glia and vascular cells and are characterized by a variety of distinct properties. One intriguing issue is how these P2X receptors are activated by the natural agonist, ATP. Our understanding of ATP signaling was recently enhanced by the reports of the X-ray crystal structures of zebrafish P2X4 receptors in the closed and open states. Arising from these results, molecular dynamics studies of receptor structure are becoming an important tool for many studies of the molecular conformation of P2X receptors, as reviewed by Chataigneau et al. (2013). The mechanism of ion channel opening, which is indispensable for P2X function, as well as the properties of ion flow through it are presented by Samways et al. (2014). The next review explains, in detail, the process of assembling P2X subunits as either homo-trimers or hetero-trimers, with consequently major changes in their functional and pharmacological properties that depend on the final subunit composition (Saul et al., 2013). The trafficking and targeting of P2X receptors to the cell membrane, which are essential steps to express their function, are discussed by Robinson and Murrell-Lagnado (2013). Once inserted into the membrane, the activity of P2X receptors is modulated by phospholipids that play a substantial role in the receptor post-translational modifications (Bernier et al., 2013).

One unusual receptor property is the gradual formation of an increasingly larger membrane ion pore during the sustained activation of some ATP-gated P2X receptors (Rokic and Stojilkovic, 2013). This phenomenon is typically observed in the P2X7 subtype, but it can also be manifested in P2X2, P2X2/X5, and P2X4 receptors. In addition to the development of certain biophysical characteristics, this property is associated with important functions, including the release of powerful immunologically-relevant molecules.

In the current e-book collection, several reviews present a detailed analysis of the mechanisms and pathophysiological role of certain receptor subtypes. Thus, one review deals with the strong desensitization properties of P2X3 receptors (Giniatullin and Nistri, 2013). Desensitization (temporary inactivation of the channels by persistent agonist application) likely plays a filtering effect in the detection of physiological nociceptive signals induced by ATP. Abnormal pain signaling via P2X3 receptors is thought to contribute to neuronal sensitization and chronic pain, whose molecular mechanisms are amply discussed in the review by Fabbretti (2013). The therapeutic applications of P2X3 receptor antagonists to pathological states are subsequently discussed by Ford and Undem (2013).

The present e-book also contains reviews on fundamental aspects of P2X receptor translational application that includes new pharmacological approaches to target acute and chronic pain, and neurological and vascular diseases. Thus, the review on the role of ATP receptors in status epilepticus and excitability of mammalian brain neurons provides novel information on the control of cortical network activity (Henshall et al., 2013). A new function of ATP signaling has emerged in taste buds where, by acting on desensitizing P2X3 subunits co-assembled with non-desensitizing P2X2 subunits on receptor cells, ATP serves as a messenger between taste buds and afferent nerves (Kinnamon and Finger, 2013).

A detailed analysis of P2X4 receptors, which together with P2X7 receptors, are expressed by glial cells, indicates their contribution to neuroinflammation and chronic pain (Tsuda et al., 2013). The companion original article presents new data on the structural and functional properties of the rat P2X4 receptor extracellular vestibule during the gating process (Rokic et al., 2014). Furthermore, an important new role for P2X4 receptors as modulators of lung surfactant secretion has been recently identified (Miklavc et al., 2013). The dynamic micro-organization of P2X7 receptors revealed by PALM based single-particle tracking is elucidated in the original contribution by Shrivastava et al. (2013).

The field of P2X receptor studies is rapidly expanding as novel drugs based on their ability to selectively antagonize P2X receptor subtypes are developed. Furthermore, mechanisms of phenomena like large pore opening or desensitization, albeit incompletely understood, may represent future targets for designing specific new drugs. The extensive survey of P2X receptors found in the present e-book allows fast perusing of specific mechanisms related to distinct receptor subtypes, and represents a solid reference for future studies. We believe that this e-book is an excellent introduction for any scientist interested in the basic and clinical role of ATP receptors, and is also a very useful source of concentrated updated information for professionals.

## Conflict of interest statement

The authors declare that the research was conducted in the absence of any commercial or financial relationships that could be construed as a potential conflict of interest.
